# Supplementary material for: Splice-Junction-Based Mapping of Alternative Isoforms in the Human Proteome
Source: Cell Rep. Author manuscript; Available in PMC 2020 Jan 15. (PMC6961840; doi:10.1016/j.celrep.2019.11.026)
Supplement: 2 [file NIHMS1546469-supplement-2.zip › DF1/PXD010154/Thyroid/Thyroid_43_TRAPPC3_IEDNLPAGELFSNFLPR.pdf]

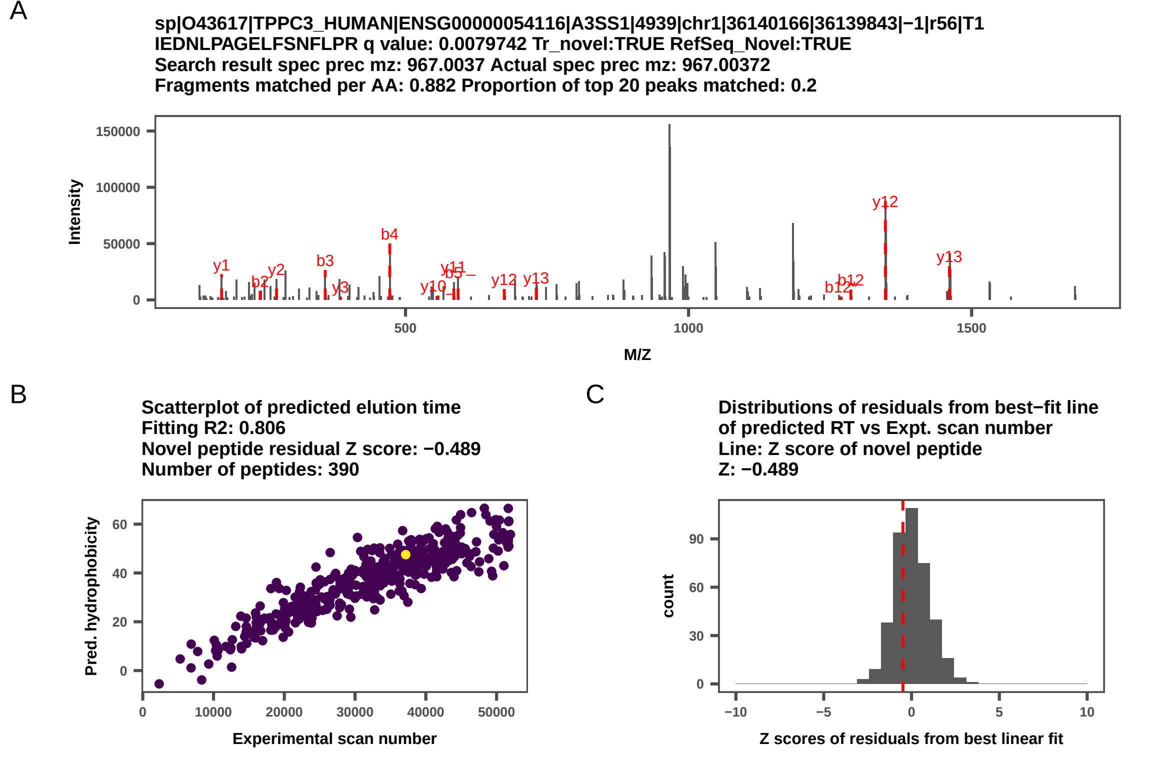
A
sp|O43617|TPPC3_HUMAN|ENSG00000054116|A3SS1|4939|chr1|36140166|36139843|−1|r56|T1
IEDNLPAGELFSNFLPR q value: 0.0079742 Tr_novel:TRUE RefSeq_Novel:TRUE
Search result spec prec mz: 967.0037 Actual spec prec mz: 967.00372
Fragments matched per AA: 0.882 Proportion of top 20 peaks matched: 0.2
Intensity
150000
100000
50000
0
y1
b2
y2
b3
y3
b4
y10
y11
b5
y12
y13
b12
b12
y12
y13
500
1000
1500
M/Z
B
Scatterplot of predicted elution time
Fitting R2: 0.806
Novel peptide residual Z score: −0.489
Number of peptides: 390
Pred. hydrophobicity
60
40
20
0
0
10000
20000
30000
40000
50000
Experimental scan number
C
Distributions of residuals from best−fit line
of predicted RT vs Expt. scan number
Line: Z score of novel peptide
Z: −0.489
count
90
60
30
0
−10
−5
0
5
10
Z scores of residuals from best linear fit
